# Neurological Abnormalities in Full-Term Asphyxiated Newborns and Salivary S100B Testing: *The “Cooperative Multitask against Brain Injury of Neonates” (CoMBINe) International Study*

**DOI:** 10.1371/journal.pone.0115194

**Published:** 2015-01-08

**Authors:** Diego Gazzolo, Francesca Pluchinotta, Moataza Bashir, Hanna Aboulgar, Hala Mufeed Said, Iskander Iman, Giorgio Ivani, Alessandra Conio, Lucia Gabriella Tina, Francesco Nigro, Giovanni Li Volti, Fabio Galvano, Fabrizio Michetti, Romolo Di Iorio, Emanuela Marinoni, Luc J. Zimmermann, Antonio D. W. Gavilanes, Hans J. S. Vles, Maria Kornacka, Darek Gruszfeld, Rosanna Frulio, Renata Sacchi, Sabina Ciotti, Francesco M. Risso, Andrea Sannia, Pasquale Florio

**Affiliations:** 1 Department of Maternal, Fetal and Neonatal Medicine, *“C. Arrigo”* Children’s Hospital Alessandria, Italy; 2 Department of Cardiology and Laboratory Reasearch S. Donato Milanese University Hospital, Milan, Italy; 3 Department of Neonatology, Cairo University, Cairo, Egypt; 4 Pediatric Intensive Care Unit, *“Regina Margherita”* Children’s Hospital Turin, Turin, Italy; 5 Department of Maternal Fetal and Neonatal Health *“G. Garibaldi”* Hospital, Catania, Italy; 6 Department of Biochemistry, Catania University, Catania, Italy; 7 Institute of Anatomy and Cell Biology, Catholic University, Rome, Italy; 8 Laboratory of Perinatal Medicine, Department of Obstetrics and Gynecology, University *“La Sapienza”*, Rome, Italy; 9 Department of Pediatrics and Neonatology, Maastricht University, Maastricht, The Netherlands; 10 Department of Child Neurology, Maastricht University, Maastricht, The Netherlands; 11 Department of Neonatology and Intensive Care of Neonate, Warsaw University, Warsaw, Poland; 12 Department of Pediatrics *“G. Gaslini”* Children’s University Hospital, Genoa, Italy; 13 UOC of Obstetrics and Gynecology, “S. Iacopo” Hospital, Pistoia, Italy; Emory University School of Medicine, UNITED STATES

## Abstract

**Background:**

Perinatal asphyxia (PA) is a leading cause of mortality and morbidity in newborns: its prognosis depends both on the severity of the asphyxia and on the immediate resuscitation to restore oxygen supply and blood circulation. Therefore, we investigated whether measurement of S100B, a consolidated marker of brain injury, in salivary fluid of PA newborns may constitute a useful tool for the early detection of asphyxia-related brain injury.

**Methods:**

We conducted a cross-sectional study in 292 full-term newborns admitted to our NICUs, of whom 48 suffered PA and 244 healthy controls admitted at our NICUs. Saliva S100B levels measurement longitudinally after birth; routine laboratory variables, neurological patterns, cerebral ultrasound and, magnetic resonance imaging were performed. The primary end-point was the presence of neurological abnormalities at 12-months after birth.

**Results:**

S100B salivary levels were significantly (P<0.001) higher in newborns with PA than in normal infants. When asphyxiated infants were subdivided according to a good (Group A; n = 15) or poor (Group B; n = 33) neurological outcome at 12-months, S100B was significantly higher at all monitoring time-points in Group B than in Group A or controls (P<0.001, for all). A cut-off >3.25 MoM S100B achieved a sensitivity of 100% (CI_5-95%_: 89.3%-100%) and a specificity of 100% (CI_5-95%_: 98.6%-100%) as a single marker for predicting the occurrence of abnormal neurological outcome (area under the ROC curve: 1.000; CI_5-95%_: 0.987-1.0).

**Conclusions:**

S100B protein measurement in saliva, soon after birth, is a useful tool to identify which asphyxiated infants are at risk of neurological sequelae.

## Introduction

Perinatal asphyxia (PA), defined as clinical situation of damaging acidemia, hypoxia, metabolic acidosis and multi organ failure in a newborn infant, is well known to cause physical harm to the brain. Brain damage is of most concern and perhaps the least likely to heal either quickly or completely since, depending on the degree of PA; it may be mental, appearing as developmental delay or intellectual disability, or physical, such as spasticity [1, 2]. Neurological handicap has been found in about 25–28% of these infants due to the early cell death resulting from primary exhaustion of the cellular energy stores and to neuronal injury occurring several hours after the initial insult caused by oxygen free radicals, intracellular calcium entry and apoptosis [2]. Even when accurate postnatal monitoring procedures are performed (i.e. determination of blood pH, measurement of uric acid and lactate, cerebral ultrasound and continuous EEG recordings) the post-asphyxia period is crucial, since brain damage may already be at a sub-clinical stage and its symptoms hidden by the effects of the therapeutic strategies adopted [3–6]. In this setting, the measurement of quantitative parameters able to diagnose sub-clinical lesions at stages when routine brain-monitoring procedures are still silent could be especially useful.

S100B protein is an acidic calcium-binding protein concentrated mainly in glial cells of the nervous system [7–11]. Early and severe central nervous system (CNS) damage is associated with continuous S100B protein release, whose concentration is detectable in different biological fluids [8–11]. There is well-documented evidence of a correlation between the extent of brain damage due to hypoxia/asphyxia and elevated concentrations of S100B [7–12], so that an increase in S100B protein level is considered an early index of CNS damage [7–17], particularly in the field of perinatal medicine [7–10].

S100B protein is also detectable in the saliva of healthy preterm and term newborns, and levels progressively decrease according to gestational age [18], with a secretory pattern resembling that already described in cord blood [19] and urine [20] of newborns. The detection of S100B in the saliva has opened new possibilities for the assessment of this protein in perinatal medicine, and the purpose of the International *“Cooperative Multitask against Brain Injury of Neonates”* (CoMBINe) Study was therefore to investigate whether the measurement of S100B levels in salivary fluid collected from asphyxiated full-term newborns may constitute a useful tool for the early detection of neurological abnormalities. The clinical performance of S100B assessment for the early prediction of poor neonatal outcome was later compared to the Score for Neonatal Acute Physiology-Perinatal Extension (SNAP-PE II), an empirically system scoring the severity of illness and mortality risk for newborn intensive care, found to be increased in PA infants in the first 12-h after birth [21].

## Materials and Methods

Forty-eight consecutive neonates with PA, who were born in our tertiary referral centers for Neonatal Intensive Care Units (NICUs), between July 2004–October 2007, were included in the study. Local Ethics Committees approved the study protocol and informed and signed consent was obtained from all parents of patients.

The Local Ethic Committees of the CoMBINe International Study (Alessandria, Turin, Catania, Rome, Genoa, Pistoia, Italy; Cairo, Egypt; Maastricht, The Netherlands; Warsaw, Poland) approved the study protocol and informed and signed consent was obtained from all parents of patients.

All asphyxiated infants were delivered by emergency caesarean section (CS) because of acute fetal distress classified in agreement with the criteria of the American College of Obstetricians and Gynecologists [22]. Asphyxia was defined as an Apgar score <3 at the 5th minute, pH <7.0, BE*<*-12 in cord or venous blood taken from newborns within 60 minutes of birth, the need for resuscitation at birth and/or for positive pressure ventilation (>3 minutes) and the occurrence of multiorgan failure [22]. Infants fulfilling 3 or more of the above criteria were included in the asphyxia group, received mechanical ventilation and were sedated by means of fentanyl citrate (Fentanest; Pharmacia & Upjohn International, Milan, Italy), 0.5 to 2.5 μg/kg per hour, and midazolam hydrochloride (Ipnovel; Roche SA, Fontenay-sous-Bois, France) 50 to 400 μg/kg per hour.

The Reference Group included healthy term neonates delivered consecutively either by elective CS (n = 54; 22.1%) or vaginally (n = 190; 77.9%), that fulfilled the following criteria: no signs of fetal distress, pH >7.2 in cord blood or venous blood, Apgar scores at 1–5 minutes >7. This decision was based on the number of deliveries at our centers (about 15,000 per year) and epidemiological studies relating to the incidence of asphyxia in the countries involved [1, 23, 24]. Exclusion criteria were: CNS malformations, chromosomal abnormalities, congenital heart disease, multiple pregnancies, congenital infections, maternal drug addiction, hypertension, and diabetes. Infants with any malformation, intrauterine growth retardation, cardiac or hemolytic disease were also excluded from the study.

Clinical and laboratory parameters were recorded in all infants on admission to NICUs and at 24 and 72-h from birth for the standard clinical assessment. SNAP-PE II score was assessed for evaluating the severity of illness within first 12-h after birth [21]. Cerebral ultrasound (US) and neurological patterns were assessed by a single examiner in each Centre, who did not know the results of the saliva test.

### Cranial Assessment

US recordings were performed by real-time ultrasound machine (Acuson 128SP5 Mountain View CA, USA), at 12 and 24-h from admission and on discharge from the hospital. In the controls, US patterns were evaluated before discharge from the hospital.

### Magnetic Resonance Imaging (MRI)

MRI was performed in asphyxiated newborns within 7-d from birth on a 1.5-T scanner. Standard sequences included sagittal and axial spin-echo T1, double-acquisition axial fast-spin echo T2 proton density, coronal fast-spin echo T2, and axial diffusion-weighted images. A single examiner in each center who did not know the results of the saliva test and clinical data reviewed images.

Images were scored according to the scheme of Barkovich et al. [25], with BG/W score (basal ganglia/watershed areas) that combines basal ganglia and watershed patterns of damage assigning a score of 0–4. Patients were classified by severity as follows: normal (0); abnormal signs in basal ganglia or thalamus (1); abnormal signs in cortex (2); abnormal signs in cortex and basal nuclei (basal ganglia or thalami) (3); abnormal signs in entire cortex and basal nuclei (4).

### Neurological examination

Neurological examination was performed at birth and at 12, 24 and 72-h of age. Neonatal neurological conditions were classified as described by Prechtl [26, 27]. Each infant was assigned to 1 of 3 diagnostic groups: normal, suspect or abnormal, in accordance with the classification used by Jurgens–van der Zee et al. [28]. An infant was considered to be abnormal when one or more of the following neurological syndromes was unequivocally present: *i)* increased or decreased excitability (hyperexcitability syndrome, convulsions, apathy syndrome or coma); *ii)* increased or decreased motility (hyperkinesia or hypokinesia); *iii)* increased or decreased tonus (hypertonia or hypotonia); *iv)* asymmetries (peripheral or central); *v)* defects of the CNS and; *vi)* any combination of the above. When indications of the presence of a syndrome were inconclusive or if only isolated symptoms were present, e.g. mild hypotonia or only a slight tremor, the case was classified as suspect.

The presence within the first 7-d from birth of hypoxic ischemic encephalopathy (HIE) was classified according to Sarnat and Sarnat [29]. HIE was defined as *“mild”* if hyperexcitability or hypotonia persisted without seizures for at least 72-h after birth; *“moderate”* if the infant was lethargic and had hypotonia, weak primitive reflexes and seizures; and *“severe”* if the infant suffered frequent seizures, apnea, flaccid weakness or coma.

EEG traces were recorded in the asphyxiated infants within 7-d from birth.

### Neurological outcome at follow-up

Neurological outcome was assessed at 12-month from birth, according to Amiel-Tison’s criteria [30], by testing tone and posture, resistance against passive movements (i.e. approximation of heel to ear, scarf sign; measurement of angles of certain joints, such as the popliteal angle), visual pursuit, reaching and grasping, and responses to visual and acoustic stimuli. The infants were scored as normal or abnormal according to the results obtained in relation to the age in months. In particular, the examiner circled a score of 0, 1, or 2, according to the information given in the technical descriptions for each maneuver. A score of 0 indicated a typical result for that age, within the normal range; a score of 1 indicated a moderately abnormal result for that age and a score of 2 indicated a definitely abnormal result. Infants with PA were divided into those with normal or adverse neurological outcome according to whether neurological abnormalities had developed at the 12-month follow-up.

### S100B measurement

Saliva samples (100 µL) were collected at the predetermined monitoring time-points by a standard collector (Pennine Heathcare, London, UK). Since S100B protein has been found at high concentrations in milk [8] caution is needed at sampling procedure in order to avoid potential bias due to S100B extra-source such as milk. This limitation was not met during the sampling procedure since none of the PA infants were fed due to their critical conditions. Identically, in healthy controls no limitations at sampling procedure were met collecting saliva every 4-h and far from feeding.

Saliva S100B levels were measured at birth (time 0) and at 4 (time 1), 8 (time 2), 12 (time 3), 16 (time 4), 20 (time 5), 24 (time 6), 48 (time 7), 72 (time 8) and 96 (time 9) hours from birth. After collection, saliva samples were immediately centrifuged at 900 g for 10 minutes and stored at -70°C until the assay. S100B protein levels were measured using the LIAISON immunoluminometric assay (Lia-mat Sangtec 100, AB Sangtec Medical, Bromma, Sweden), according to the manufacturer’s instructions. Each measurement was performed in duplicate and averages were reported. The detection limit of the assay was 0.02 µg/L, the coefficient of variation was 3.9% or lower within-assay and 6.2% or lower inter-assay for concentrations ranging between 0.12 and 17.5 µg/L.

## Statistical Analysis

As S100B protein levels changes with gestational age [20] concentrations were corrected for gestational age at birth by conversion to multiples of median (MoM) of healthy controls of the same gestational age interval (every two weeks (37–38 wks, n = 102; 39–40 wks, n = 67; 41–42 wks, n = 75): 37–38 wks, n = 102; median: 0.5; minimum: 0.02; maximum: 1.3. 39–40 wks, n = 67; median: 0.40; minimum: 0.03; maximum: 1.3. 41–42 wks, n = 75; median: 0.23; minimum: 0.01; maximum: 0.55). The Reference Group was thus composed by healthy infants stratified by gestational age and the medians of each stratum were used to convert all values to MoM. Infants were matched with control medians according to gestational age at the time of salivary fluid sampling. S100B (µg/L) concentrations were divided by the median values of control groups belonging to the same gestational interval medians of each. The MoM conversion improves statistical power and permits external validation of results from different populations, besides allowing regression analysis of data in order to reach a straight estimation of the expected normal S100B concentrations.

When the Kolmogorov-Smirnov test showed that values were not normally distributed, S100B concentrations were expressed as medians and ranges [lower and upper 95% Confidence Interval (CI)] and statistical significance of differences evaluated by using non-parametric test. Data on neonatal outcomes and laboratory parameters were analyzed according to Tukey’s one-way ANOVA and the two-sided Mann-Whitney U-test. Comparison between proportions was performed using Fisher’s exact test. Sensitivity, specificity and predictive values of S100B and SNAP-PE as diagnostic tests for the detection of brain damage in PA newborns were assessed using the Receiver Operating Characteristic curve (ROC) test [31]. The probability of developing brain damage when no, one, or both tests were positive (higher than the cut-off point) was estimated and compared with the pre-test probability, defined as the prevalence of brain damage in the whole group of newborns [32]. Statistical significance was set at P<0.05.

## Results

### Neonatal outcomes and clinical findings

Table 1 shows neonatal outcomes and clinical characteristics of the neonatal population evaluated at the different monitoring times and at 12-month. Four out of the 48 asphyxiated infants died due to multi-organ failure between 72 and 96-h from birth and therefore were skipped from further analysis. At the 12-month follow-up (see below), asphyxiated neonates were assessed as either normal (n = 15; Group A) or adverse (n = 29; Group B) neurological outcome.

**Table 1:** Perinatal clinical characteristics and outcomes in asphyxiated newborns with normal (Group A) or abnormal (Group B) neurological outcome and in healthy subjects (Reference Group, R Group).

	**Group A (n = 15)**	**Group B (n = 29)**	**R Group (n = 244)**
Perinatal Clinical Characteristics			
Birth weight (g)	3,466 ± 216	3,354 ± 299	3,371 ± 312
Gestational age >36 wks (no.)	15	29	244
Gender (male/female)	8/5	14/15	101/143
Caesarean Section (no./total)	15/15^*^	29/29^*^	54/244
Factors associated with primary outcomes			
Apgar score at 1 min <3 (no./total)	15/15^*^	29/29^*^	0/244
Apgar score at 5 min <3 (no./total)	15/15^*^	29/29^*^	0/244
**Respiratory distress syndrome (no./total)**	2/15^*^	11/29^*^	0/244
Mechanical ventilation support (no/total)	2/15^*^	11/29^*^	0/244
Inotrope therapy (no./total)	12/15^*^	23/29^*^	0/244
SNAP-PE score	47.1 ± 7.4^*^	51.9± 7.8^*^	41.6 ± 6.5
Cerebral US normal/hyperechogenicity/bleeding (no.)			
at birth	15/0/0	29/0/0	n.p.
at 12 h	15/0/0	29/0/0	n.p.
at 24 h	8/6/1^*^	15/10/4^*^	n.p.
at 72 h	8/6/1^*^	15/10/4^*^	244/0/0
HIE mild/moderate/severe (no.)	3/9/3^*^	1/3/25^*^	244/0/0
Prechtl’s test results: normal/suspect/abnormal (no.)			
at birth	6/9/0^*^	7/9/13^*^	244/0/0
at 12 h	6/9/0^*^	7/9/13^*^	244/0/0
at 24 h	6/9/0^*^	7/9/13^*^	244/0/0
at 72 h	6/9/0^*^	7/9/13^*^	244/0/0
Amiel-Tison test results at 12 months follow-up normal/suspected/abnormal (no.)	15/0/0	0/0/29^*^	244/0/0

Values are expressed as mean ± SD. n.p.: not performed.

^*^P<0.01 vs controls.

No significant differences were found in asphyxiated and control groups regarding maternal age, gestational age and weight at birth or gender (P>0.05), whilst the incidence of emergency caesarean sections (P<0.01) was significantly higher in the asphyxiated infants than in controls (P<0.05). No previous coagulopathies were found in infants participating to the study. As expected, Apgar scores at 1–5 minutes, as well as the incidence of acute RDS, the need for mechanical ventilation support and inotrope therapy, the occurrence of multiorgan failure (Group A: n = 0; Group B: n = 12) differed significantly (P<0.05, for all) between the asphyxiated (Groups A and B) and healthy (Reference Group) neonates (Table 1). Again, the mean (SD) SNAP-PE score recorded within the first 24-h after birth was significantly lower in healthy controls (41.6 ± 6.5) than in asphyxiated newborns with good (Group A: 47.1 ± 7.4; P<0.01), or severe (Group B: 52.3 ± 9.8; P<0.001) neurological prognosis, while not differing between the two groups with perinatal asphyxia (Table 1).

The incidence of HIE and of abnormal neurological examination results, and the presence of abnormal cerebral US patterns were significantly (P<0.05) higher in the asphyxiated groups than in controls. Indeed, moderate HIE was observed in 12 asphyxiated neonates (Group A: n = 9; Group B; n = 3) and severe HIE was present in 32 infants (Group A: n = 3; Group B: n = 29) (Table 1).

US patterns suggestive of brain edema and/or periventricular hyper-echogenicity were observed, in 16 out of 48 infants with PA, at 24-h from birth (Table 1). At the 24-h and 72-h monitoring time-points five asphyxiated infants showed positive patterns for cerebral bleeding [Group A: n = 1 intraventricular Hemorrhage (IVH); Group B: n = 4 IVH with ventricular dilation]. Neonates with IVH in Group B later died [at 74 (n = 2) and 96 (n = 2) hours from birth]: 3 of them had sub-ependymal hemorrhage, the fourth infant developed IVH with ventricular dilatation. None of the monitored infants showed congenital cerebral malformations and identical cerebral US patterns were observed 72-h after NICU admission.

MRI was performed in all surviving infants within 7-d from birth. Intra-observer variability was assessed in all PA infants showed a variability of 2.5% (P>0.05). No overt differences in MRI and in cerebral US patterns were detectable regarding cerebral bleeding characteristics in asphyxiated infants. MRI injury was present in 29 patients. BW/G score was significantly higher (P<0.05) in Group B than Group A.

According to Prechtl’s evaluation of neonatal neurological conditions on admission and at different monitoring time-points, 18 of 48 asphyxiated infants were classified as suspect [Group A: hypo/hypertonia (n = 5), hyper-excitability (n = 4); Group B: hyper-excitability (n = 4); hypo/hypertonia (n = 5)] (Table 1). However, on account of their severe clinical conditions, the effects of sedative drugs and intervention by NICUs, neurological examination was inconclusive, especially during the first 24-h after asphyxia insult. No abnormal Prechtl’s test result was found in Group A.

The results of neurological examination at 12-month were normal in Group A and control neonates, whereas neurological abnormalities in Group B at this time included hypo/hypertonia syndrome (n = 15), hemisyndrome (n = 9) and quadriparesis (n = 5).

### Laboratory findings

Table 2 shows the laboratory parameters recorded at birth. Venous blood pH, partial venous CO_2_ and O_2_ pressure and base excess values differed significantly between asphyxiated and control groups (P<0.01), whilst no significant differences were observed among groups regarding red blood cell count, hemoglobin concentration, hematocrit and blood concentration of ions (P>0.05). There were no significant differences in perinatal data and laboratory parameters observed between the sub-groups of asphyxiated infants (P>0.05) (Table 2). Identical patterns were observed between the sub-groups of asphyxiated infants in laboratory and monitoring parameters at any of the remaining monitoring time-points (data not shown).

**Table 2:** Laboratory parameters recorded at birth in asphyxiated newborns with normal (Group A) or abnormal (Group B) neurological outcome and in healthy subjects (Reference Group, R Group).

	**Group A (n = 15)**	**Group B (n = 33)**	**R Group (n = 244)**
Venous blood pH	7.03 ± 0.01^*^	7.00 ± 0.02^*^	7.36 ± 0.06
Partial venous CO2 pressure (mmHg)	69.6 ± 1.8^*^	66.3 ± 4.9^*^	41.3 ± 0.5
Partial venous O2 pressure (mmHg)	21.1 ± 0.9^*^	19.1 ± 1.7^*^	40.7 ± 0.6
Base excess	-13.5 ± 0.2^*^	-13.1 ± 0.3^*^	-0.2 ± 1.1

Values are expressed as mean ± SD.

^*^P<0.0001 vs controls.

### S100B measurement

**Figure 1:**
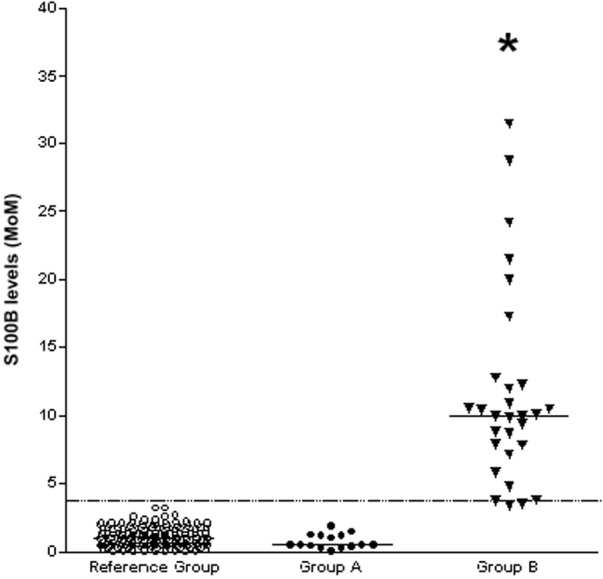
Saliva levels of S100B were significantly higher in asphyxiated full-term newborns with severe (Group B, black triangle) than in those with good (Group A, black circles) neurological outcome at follow-up and in healthy controls (Reference Group, open circles). The horizontal solid lines indicate the median values for each group; the horizontal dotted line indicates the cut-off point (critical value) with the most reliable separation between infants who suffered severe long-term neurological sequelae and those who did not and controls. *P<0.001 vs healthy controls and asphyxiated newborns with absent or mild HIE.

S100B was detected in all the saliva samples collected at birth and at subsequent monitoring time-points. As expected, S100B levels were found to decrease significantly between the 37th-38th and 41^st^–42^nd^ weeks of gestation in healthy controls (20) (P<0.001, data not shown). When corrected for gestational age and converted to MoM, S100B levels in saliva at birth were highest in Group B (mean 11.6 MoM; CI 8.9–14.4 MoM), and significantly higher than both controls (Reference Group; mean 1.1 MoM; CI 1.02–1.2 MoM; P<0.001) and Group A (mean 0.8 MoM; CI 0.5–1.08 MoM; P<0.001): however, no significant difference was found between Group A and controls (Fig. 1).

Table 3 shows S100B levels in samples of salivary fluid collected from birth to 96-h after birth h (time 0–9). Levels did not change during monitoring in either healthy neonates or asphyxiated newborns with good prognosis (Group A), but in asphyxiated newborns with severe neonatal outcome (Group B) they were higher at time-points 0–6, decreasing to reference values after 48-h from birth (P<0.001) (Table 3). In these infants, S100B concentrations were significantly higher at monitoring time-points 0–6 (P<0.001 for all) than in both healthy and Group A infants (P<0.001), whilst they did not differ between these latter two groups (Table 3). The 4 infants who died at 74 and 96-h after birth showed the highest saliva S100B concentrations from first collection (19.8; 40.0; 41.1; 79.6 MoM, respectively) and the levels remained higher at all monitoring times in Group B infants than in Group A or controls, whether or not the 4 infants who died in the postnatal period were included in Group B (P<0.01). Furthermore, a significant difference in S100B levels has been found between asphyxiated infants with or without seizures (P<0.05, data not shown) [14].

**Table 3:** Mean saliva S100B concentrations (µg/mL) expressed as MoM [lower and upper 95% Confidence Interval (CI)] at birth (T0) and at 4 (T1), 8 (T2), 12 (T3), 16 (T4), 20 (T5), 24 (T6), 48 (T7), 72 (T8) and 96 (T9) hours after birth in Reference Group (n = 244) and in asphyxiated full-term newborns with good (Group A) or severe (Group B) neurological outcome at 12-months follow-up.

**S100B (MoM)**	**Reference Group (n = 244)**			**Group A (n = 15)**			**Group B (n = 33)**		
	***Mean***	***Lower CI_95%_***	***Upper CI_95%_***	***Mean***	***Lower CI_95%_***	***Upper CI_95%_***	***Mean***	***Lower CI_95%_***	***Upper CI_95%_***
T0	1.10	1.02	1.20	0.80	0.50	1.08	23.6^*^	9.80	37.40
T1	1.00	1.01	1.10	1.00	0.90	1.10	26.4^*^	10.20	38.60
T2	1.00	1.01	1.10	1.00	0.90	1.10	26.4^*^	10.20	38.60
T3	1.10	1.02	1.20	0.80	0.60	1.00	24.8^*^	10.80	39.60
T4	0.98	0.96	1.00	1.00	0.90	1.10	23.8^*^	9.60	37.60
T5	1.00	0.9	1.10	1.10	1.02	1.20	23.0^*^	9.00	34.50
T6	1.10	1.02	1.20	1.10	1.02	1.20	18.2^*^	4.20	22.10
T7	1.10	1.02	1.20	1.10	1.02	1.20	1.00	0.90	1.10
T8	1.10	1.02	1.20	1.00	0.90	1.10	0.80	0.60	1.00
T9	1.10	1.02	1.20	1.00	0.90	1.10	1.10	1.02	1.20

^*^P<0.001 vs Healthy Group and asphyxiated full-term newborns with good neurological prognosis (Group A).^*^P<0.001 vs Group B values at 48, 72 and, 96 hours

Salivary S100B concentrations were significantly higher in neonates belonging to Group B at all monitoring time-points (p<0.001, for all).

Statistical evaluation of differences among Groups at each time point was performed by using the Kruskal-Wallis test followed by the Dunn’s post test

**Figure 2:**
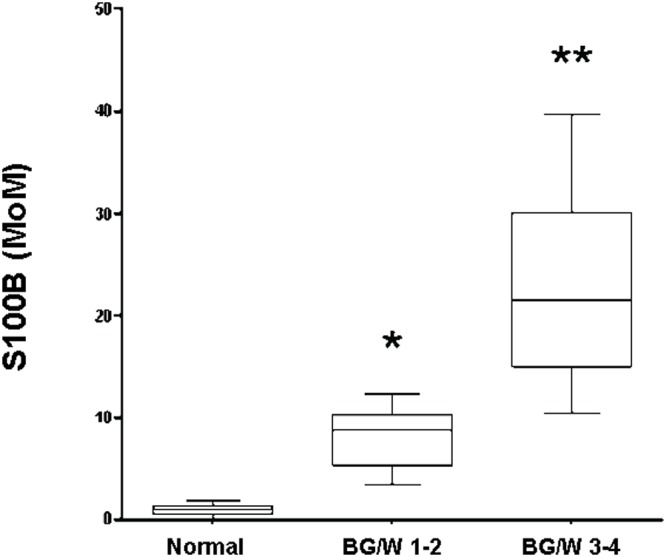
Saliva levels of S100B in neonates with normal, BG/W score 1–2 and BG/W score 3–4. The box plots represent the medians and interquartile ranges for each group. ^*^^*^P<0.001 global BG/W vs severe BG/W injury; ^*^P<0.001 vs normal MRI group.

Fig. 2 refers to S100B concentrations in neonates according to severity of MRI findings. In details, since saliva S100B concentrations were significantly highest from first collection in newborns belonging to Group B (Table 3), we used values collected at such a time-point to do comparisons. Levels were significantly (P<0.001) higher in patients with BG/W score 3–4 than in those with BG/W score 1–2 and normal MRI (P<0.001). Finally, concentrations of S100B were also significantly (P<0.001) higher in newborns with BG/W score 1–2 than those with normal MRI.

At the cut-off >3.25 MoM, chosen by the ROC curve analysis, S100B at birth achieved a sensitivity of 100% (CI: 89.3–100%) and a specificity of 100% (CI: 98.6–100%) as a single marker for predicting the occurrence of poor neurological outcome [area under the ROC curve (AUC): 1.000; CI: 0.987–1.0) (Fig. 1). In contrast, SNAP-PE scores at the cut-off >53 combined a sensitivity of 69.8% (CI: 51.3–84.4%) with a specificity of 93.8% (CI: 90.2–96.4%) for predicting poor neurological outcome (AUC: 0.806; CI: 0.756–0.850). The SNAP-PE AUC was significantly (P<0.001) lower than that calculated for S100B at time 0 (SD: 0.0471; difference between AUCs: 0.194; CI: 0.102–0.286).

Twenty-nine of 48 asphyxiated infants had a poor neurological outcome at follow-up, making an overall prevalence of the long-term brain damage sequelae in our population of 60.4% (CI: 43.4–77.4%). This was the predicted probability of developing brain damage before S100B was measured (pretest probability). If only S100B was available the positive predictive value was 100.0% (CI: 98.04–100%) and the negative predictive value 100.0% (CI: 98–100%); using SNAP-PE computation, positive and negative predictive values were 42.5% (CI: 27.2–57.8%) and 95.6% (CI: 93.6–98.4%), respectively.

## Discussion

Perinatal asphyxia is a major cause of death and of acquired brain damage in newborn infants worldwide [1]: its prognosis depends on both the severity of the asphyxia and on the immediate resuscitation to restore oxygen supply and blood circulation to limit the extent of brain injury [1]. In the present study we report that soon after a severe hypoxic insult, salivary concentrations of S100B increase, in particular in asphyxiated infants with a poor short-term neonatal outcome. This results may be explained bearing in mind that: *i)* early and severe damage to the CNS is associated with the continuous release of S100B protein which is present and measurable in the bloodstream [33, 34], and; *ii)* hypoxia/asphyxia triggers S100B release, so that raised protein’ concentrations have been interpreted as a direct indicator of active neuronal cell damage [14, 16–19, 35–37].

This hypothesis is sustained by the evidences that S100B is absent from human fetal salivary glands tissue from 32 weeks of gestation onwards and in adulthood [38] and, therefore the increase in S100B is an event that usually occurs in the blood, cerebrospinal fluid and urine of full-term asphyxiated newborns with HIE [10, 14, 16, 17]. Therefore, these facts and findings of the present study, taken together, do suggest that: *i)* the release of S100B constitutes a warning sign of severe brain insults; *ii)* a higher release of S100B correlates with more severe brain injury, as concentrations were highest in the asphyxiated infants who developed short/long-term neurological disability than in those who did not or in controls and; *iii)* S100B concentrations in neonates vary according to the severity of MRI findings.

Another point that merits discussion is the putative clinical utility of testing S100B as early as possible at birth. Indeed, we found that in newborns with saliva S100B levels above the thresholds defined by the ROC curve analysis (>3.25 MoM), the probability (positive predictive value) of neurological sequelae was as high as 100%, while it was 0% if these levels were below the threshold, with positive and negative predictive values that differ from the overall prevalence of neonatal brain damage at 12 months (10.1%) in the study population. These findings imply the possibility of identifying newborns at higher risk of poor neurological outcome in the first hours after birth. Of note, most centres to identify patients needing therapy rely on clinical examination that may be confounded by neuroactive medications and interference from medical support devices. And, also the use of MRI and EEG data, although predictive of outcome, may not be useful immediately after birth for several reasons. Indeed, MRI has limited value in the first 24 hours of life [39] and is restricted by the impracticability of transporting a critically ill neonate for imaging, as well as, EEG data require equipment and interpretive expertise not available at many centers. On the contrary, by measuring S100B, the identification of infants at risk of long-term brain damage sequelae can be obtained at earliest stage, at a time when standard diagnostic procedures are still silent or unreliable and predicting severity of MRI findings. Anyway, further investigations in a wider cohort in order to validate the present findings are so justified.

Saliva samples are much more easily collectable than blood, cerebrospinal fluid or urine. Thus, the S100B augmentation after perinatal asphyxia event would support the expedience of performing reliable S100B measurements in saliva, in order to easily diagnose/monitor brain distress. Thanks to the short (less than 1 hour) half-life of S100B [40] it would be also possible to perform longitudinal assessment during step-by-step monitoring of asphyxiated infants. In our cases, concentrations of S100B were increased only in those infants in whom asphyxia was more pronounced and cerebral damage more severe, compared to cases affected simply by hypoxia. In other words, the persistence of high protein’s concentration up to 24 hours from birth points towards a continuous S100B release indicating the occurrence of a dramatic damage in CNS. As a consequence, S100B assessment may offer the opportunity to identify cases requiring neuroprotective strategies, thus monitoring the effectiveness/side-effects of therapeutic strategies such as selective/total body cooling, especially during the delicate phases (i.e. cooling and rewarming) [41].
